# A Comparative Study of Energy Contents in Mosquito Vectors of Malaria and Dengue Prevailing in Jodhpur City (Thar Desert) of Rajasthan State, India

**Published:** 2018-09-30

**Authors:** Suman Sundar Mohanty, Sunita Meena, Phool Chand Kanojia

**Affiliations:** 1 Desert Medicine Research Centre (Indian Council of Medical Research), New Pali Road, Jodhpur, India; 2 Department of Biotechnology, Jaipur National University, Jaipur, Rajasthan, India

**Keywords:** *Aedes aegypti*, *Anopheles stephensi*, Glucose, Glycogen, Lipid

## Abstract

**Background:**

Transmission of malaria and dengue in the desert part of India is mainly caused by *Anopheles stephensi* and *Aedes aegypti* respectively. The maintenance and transmission of the pathogens that cause malaria and dengue are dependent on the physiology of the mosquito vectors. We aimed to measure the energy contents in the mosquitoes transmitting malaria and dengue in the desert part of the country.

**Methods:**

Immature stages of mosquitoes were collected from six different larval habitats situated in Jodhpur City of Rajasthan state, India. The immature stages of both the mosquitoes were collected once in fortnightly from each location. Quantitative estimations of the lipid, glucose, and glycogen of the laboratory-reared and field collected *An. stephensi* and *Ae. aegypti* were made by spectrophotometric method. The energy contents of the larvae, pupae, females, and males were estimated in triplicates on six different occasions.

**Results:**

The lipid content of laboratory-reared larvae, pupae and female mosquitoes of *An. stephensi* and *Ae. aegypti* was found to be lower than their conspecific field-collected specimens. Whereas, the glycogen content in the laboratory-reared larvae, pupae and female mosquitoes of *An. stephensi* and *Ae. aegypti* was higher than that of their conspecific field-collected specimens. The glucose content in all the stages of the laboratory-reared *An. stephensi* was lower than their conspecific field-collected specimens except in few cases.

**Conclusion:**

The higher amount of lipid in field-collected mosquitoes may be because of the availability of food in the natural habitat and adaptation of mosquitoes. Mosquitoes living in desert climate are physiologically better equipped to survive in the desert environment.

## Introduction

*Anopheles stephensi* and *Aedes aegypti* are medically the two most important arthropod vectors of malaria and dengue respectively (1). Malaria and dengue cases are being reported from the desert zone due to introduction of canal irrigation for the development of agriculture. *Anopheles stephensi* is predominant and its presence was chronologically oldest among the entire vector species available in the desert part of India (2). The transmission of malaria in the non-irrigated part of the desert is mainly vectored by this species. It breeds in earthen pits (tanka and beri) situated in and around houses and can survive in the extremes of temperature and low humidity (3). *Aedes aegypti* is one of the most successful worldwide invaders, spreading from its native Africa to most tropical and subtropical regions of the world (4, 5) and is the primary vector of dengue fever, chikungunya, and Zika viruses.

Many dengue outbreaks have been recorded by this species, including several in Brazil and the Caribbean (6). *Aedes aegypti*, the vector of dengue is widely present in India including the Thar Desert in north-western Rajasthan (7, 8). Rajasthan is one of the dengue endemic States in India (9, 10). The maintenance and transmission of the pathogens that cause malaria and numerous viral infections are dependent on the availability of competent mosquito vectors (11).

Adult mosquitoes require carbohydrates in the form of glucose or glycogen for flight and survival during most of their adult life (12, 13). Lipid is used by the mosquitoes as energy source for long-term maintenance (14). Female mosquitoes supported by stored lipids can survive for long periods resting on the ground or in dense foliage under unfavorable nutritional and climatic conditions. In addition to energy for flight, longevity, and reproduction, nutritional status also influences the immune melanization response of *Anopheles* mosquitoes (15, 16) and susceptibility of *Aedes* mosquitoes to arbovirus (arthropod-borne virus) infection (17, 18). Newly emerged mosquitoes have different amounts of energy reserves that accumulate during their larval period (19). Some mosquitoes fail to locate blood or sugar meal after emergence and the length of the time to locate food for survival is of importance (20). Therefore, mosquitoes with higher energy reserves have a better chance of survival than the mosquitoes with lower level of the reserves.

We aimed to measure the energy contents in the mosquitoes transmitting malaria and dengue in the desert part of the country. The levels of lipid, glucose, and glycogen in field collected and laboratory-reared *An. stephensi* and *Ae. aegypti* are presented in this paper.

## Materials and Methods

### Study site

The desert zone of Rajasthan state is spread over 75000km^2^ and divided into 12 districts (2). Two seasonal rivers Luni and Mithri are present in the desert though their base is saline water. There is no perennial river flows in desert. The temperature varies from 49 °C in summer to 1 °C in winter. Immature stages of mosquitoes were collected from different larval habitat situated in Jodhpur City (Latitude: 26°18′N, Longitude: 73°04′E) of Rajasthan state, India. Immature stages were collected from the suburban and urban part of the city. The larvae were collected from Jhalamond, Kudi, Bomba Mohalla, Kheme Ka Kuan, Pratapnagar and Salawas areas of the city. The study sites were chosen on the basis of the geographic locations and availability of larval habitats.

### Sources and collection of *Anopheles stephensi* and *Aedes agypti*

Larvae and pupae of *An. stephensi* were gathered from larval habitats such as underground water storage tanks, overhead tanks, fountains and seepage areas using the standard sampling method. The larvae and pupae of *An. stephensi* were collected by strainer by the larval sampling method of Service (21). However, wrigglers were collected by droppers and mugs. *Aedes aegypti* larvae and pupae were collected from the cattle drinking waters pots, water coolers, and temporary water storage tanks. *Aedes aegypti* larvae were collected from the bottom of the storage tanks and containers through a pipe. The outlet of the pipe was opened to a bucket and immature stages of mosquitoes were collected. They were transported to the insectary in ambient condition and reared (22). The immature stages of both the mosquitoes were collected once in fortnight from each location. The larvae and pupae collected at each site were combined and placed in pans in separate cages with date and location labels. Minimum 20 collections were made from each site during the study period. Preliminary identification in the field was done visually by examining the larvae during collection and species identification was confirmed in the laboratory, after the larvae had been reared to adults, using the keys by (23–27).

### Maintenance of field-collected *Anopheles stephensi* and *Aedes aegypti*

The immature stages of *An. stephensi* and *Ae. aegypti* were collected from the field in all the seasons of the year. They were collected in containers along with the water and substrates where they were breeding. The field collected larvae and pupae were kept in the enamel trays after transported from the field. They were reared in the water and substrates collected from the breeding habitats. The food particles in water, as well as on the substrate were the source of their diet. Those larvae collected from field were not provided any additional food. They were maintained at an optimum photoperiod (10:14 [L/D] h), temperature (28±1 °C) and relative humidity (75±5%). Field collected pupae were kept in the cages for the adult emergence. Larvae, pupae, and adults of *An. stephensi* and *Ae. aegypti* were kept separately. Adults came out from the field collected pupae were kept in the cages.

### Colonization of laboratory-reared *Anopheles stephensi* and *Aedes aegypti*

The laboratory-reared *An. stephensi* colony was originated from field-collected mosquitoes of urban areas of Jodhpur, India. Similarly, *Ae. aegypti* colony used in this study was originated from field-collected mosquitoes from water containers in Bomba Mahala of Jodhpur. Both the colonies were established in 2009. In the insectary, *An. stephensi* and *Ae. aegypti* colonies were maintained at an optimum photoperiod (10:14 [L/D] h), temperature (28±1 °C) and relative humidity (75±5%). Adults were kept in the cages (0.6m^3^) and allowed to mate freely. Larvae were fed on a mixture of yeast powder and dog biscuit (1: 2) as food at 24h intervals. Overall, 10mg of the above mixture was supplied to 100 larvae per day. Soaked raisins and 10% glucose solution were provided to emerging adults. The adults were kept in cyclic cages for successive generation and maintained (22).

### Selection of mosquitoes for the assays

Fifty field-collected pupae of *An. stephensi* and *Ae. aegypti* were put separately along with the field water in the cages (0.6m^3^) for the adult emergence. Same numbers of laboratory reared pupae of both the mosquitoes were transferred to enamel bowls and kept in separate cages. Pupae of both the species were left in the cages for the adult emergence and pupae not emerged to adults were discarded on day-3. Each of ten males and females was taken for the glucose, glycogen and lipid as-says in the intervals of 24 hours. Blood meal and glucose solution was not given to the experimental female mosquitoes. Three sets of mosquitoes were taken for the tests on each occasion. Similarly, 10 larvae and pupae were taken each time and each set of experiments were conducted three times. The experiments were conducted 6 times in a year.

### Quantification of lipids, glucose, and glycogen

The contents of lipids, glucose and glycogen in field collected and laboratory-reared mosquitoes were measured by using a spectrophotometric method (28). Ten males and females were collected in the test tubes from the cages for the quantification of lipids, glucose, and glycogen. These test tubes were placed in a freezer for 5min to anesthetize the mosquitoes. Each anesthetized mosquito was transferred to a 2ml microfuge vial. Two hundred μl of 2% sodium sulfate solution was dispensed to each microfuge tube containing mosquito. Wings of the adult mosquitoes were removed before grinding. Whole mosquitoes were ground in microfuge vials by pestles for homogenization of the tissue. Similarly, each larva and pupae were homogenized in a 2ml microfuge tube containing 200μl of 2% sodium sulfate solution. Homogenized mosquito was mixed with 1.5ml chloroform-methanol solution (1: 2) and vertex for 1min. The above mixture was centrifuged at 3000rpm for 1 minute. Pellet was retained for glycogen analysis. The supernatant containing glucose and lipids was taken up with a micro-pipette and added in another microfuge of 2ml. Totally, 600μl of deionized water was added to the supernatant and mixed properly. This solution (containing sugars and lipid) was centrifuged at 3000 rpm for one minute. The lipid and sugar layers were distinctly separated. The top fraction (water/methanol) was taken up for sugar analysis and a bottom portion (chloroform) for lipid analysis. The experiments were conducted in triplicates and six times a year.

### Lipid determination

The solvent taken up from the bottom portion was evaporated completely in a heating block. Thereafter, 200μl sulphuric acid was added to the tubes and re-heated for 10min to convert the unsaturated lipids to water-soluble sulphonic acid derivatives (28). These developed a deep pink colour after addition of 5mL vanillin–phosphoric acid reagent, read in a microplate reader (Micro-Scan MS 5608) at 525nm. Lipid concentrations were obtained from a standard curve made with soybean oil. Soybean oil of 1mg was dissolved in 1ml of chloroform. Out of which 20, 50, 100, 200 and 400μL were put separately in tubes. These tubes were treated as explained above. The OD has been taken at 525nm. The standard curve was made from the average of three sets of ODs (standard as mentioned above) versus concentration.

### Glycogen determination

The precipitate in the first microfuge containing the glycogen was washed with methanol to eliminate residual sugars. Then anthrone was filled up to 5mL level and mixed properly. The tube was heated for 17min and thereafter kept for cooling. The solution was mixed and 200μL was transferred to an untreated, flat-bottomed 96-well plate. The plate was read at 625nm against the blank in microplate reader (Micro-Scan MS 5608). Glycogen concentration was calculated for individual mosquitoes from a standard curve of absorbance of known concentration of glucose as described earlier.

### Glucose determination

The top portion of the supernatant was taken up for the glucose analysis. The same was added to the tube and evaporated in a heating block till 0.1ml remained. Anthrone was filled up to 5ml level and mixed properly. The heat breaks down the body sugars into their glucose units. Anthrone binds to the glucose units and turning the mixture green. The anthrone sugar mixture was heated for 17min and left to cool (28). After cooling they were mixed properly. Overall, 200μL was transferred to an untreated, flat-bottomed 96-well plate. The plate was read at 625nm against the blank in a microplate reader (Micro-Scan MS 5608). The standard solution of glucose was prepared by dissolving 1mg of glucose in 1ml of 25% ethanol. About, 25, 50, 100, 200 and 400μl of the above glucose solution were kept in separate tubes. They were processed as mentioned above and optical densities (OD) were taken at 625nm. The standard curve was made from the average of three sets of ODs (standard as mentioned above) versus concentration. Glucose concentrations were calculated for individual mosquitoes from the standard curve of absorbance of known concentrations of glucose.

### Statistical Analysis

To analyze the habitat-specific variations between the energy levels i.e. lipid, glycogen, and glucose the data the data were subjected to two-way factorial Analysis of Variance (ANOVA). The Analysis of Variance (ANOVA) was done by using the software MS Excel software.

## Results

### Lipid

**Fig. 1.**
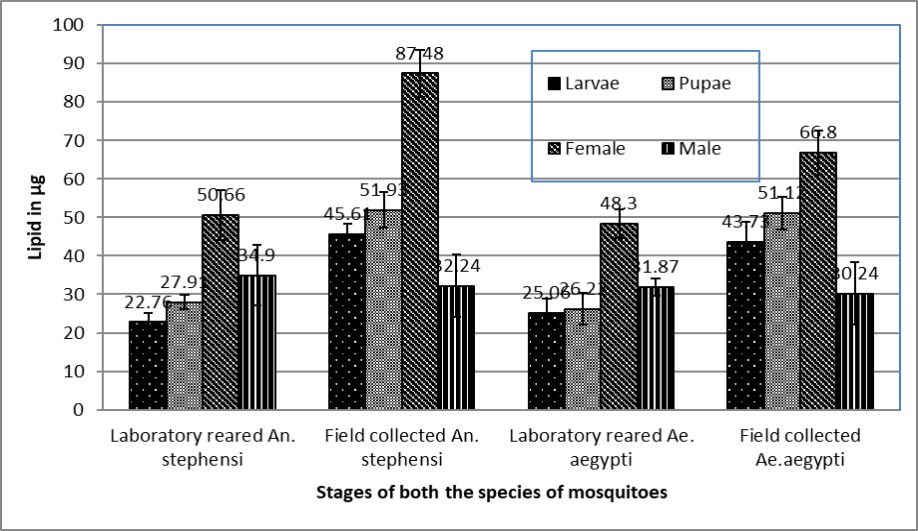
The level of lipid (mean±SE) in different stages of the laboratory-reared and field collected *Anopheles stephensi* and *Aedes aegypti*

The lipid level in both fields collected and laboratory-reared larvae, pupae, and adults of *An. stephensi* and *Ae. aegypti* is shown in Fig. 1. When the lipid contents of laboratory-reared and field-collected mosquitoes of *An. stephensi* were compared, lipid content in field-collected larvae, pupae, and female *An. stephensi* was 22.85, 24.02 and 36.82μg more than the laboratory-reared mosquitoes respectively. Similarly when the lipid content was compared between the field collection and laboratory-reared *Ae. aegypti*, lipid content in field-collected larvae, pupae and females were found to be 18.71, 24.9 and 18.5μg higher than the laboratory-reared mosquitoes respectively. The difference in the lipid content between the laboratory-reared and field-collected mosquitoes of *Ae. aegypti* was found to be highly significant (ANOVA, F= 14.24, df= 1, 3, P= 0.03). The lipid content in the males of laboratory-reared *An. stephensi* and *Ae. aegypti* was 2.66 and 1.73μg higher than the filed collected males respectively.

### Glycogen

**Fig. 2.**
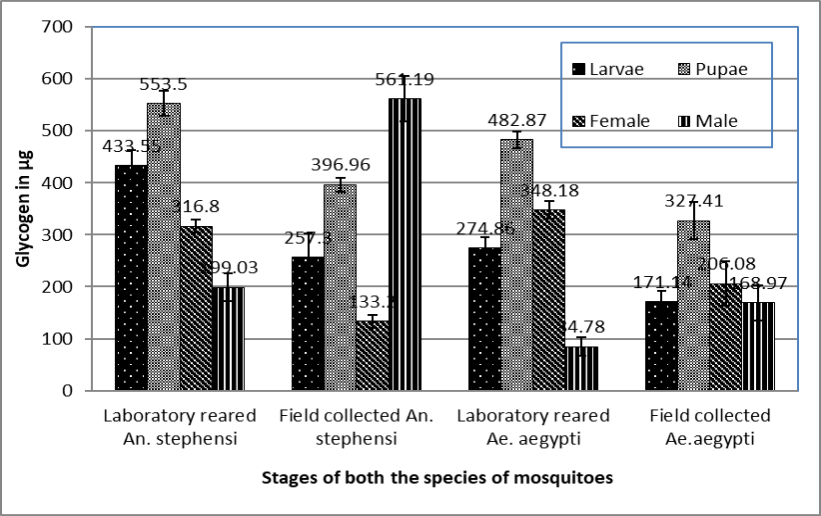
The level of glycogen (mean±SE) in different stages of the laboratory-reared and field collected *Anopheles stephensi* and *Aedes aegypti*

The concentration of glycogen content in different stages of laboratory-reared and field collected stages of *An. stephensi* and *Ae. aegypti* are shown in Fig. 2. When the glycogen content in the laboratory-reared and field collected *An. stephensi* was compared, larvae, pupae, and males of laboratory conditions were 176.25, 156.54 and 183.6μg higher than the conspecific field-collected mosquitoes (Fig. 2). Similarly, the glycogen content in the field collected larvae, pupae, and females *Ae. aegypti* were 103.72, 55 and 142.1 μg less than the conspecific laboratory reared *Ae. aegypti*. The glycogen contents in the males of laboratory-reared *An. stephensi* and *Ae. aegypti* was 362.16 and 84.19μg lower than the field collected males.

### Glucose

**Fig. 3.**
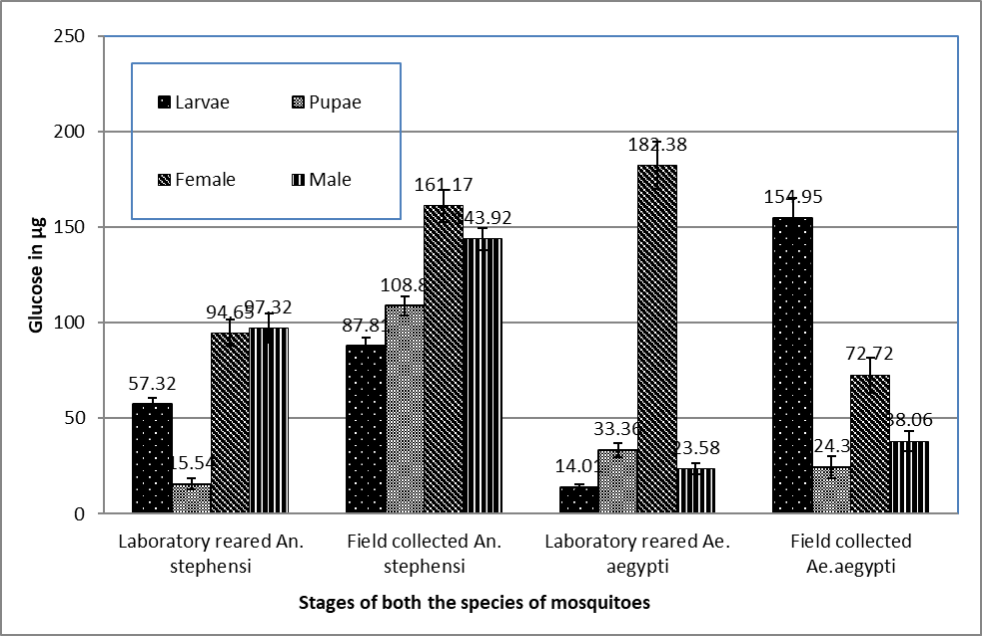
The level of glucose (mean±SE) in different stages of the laboratory-reared and field collected *Anopheles stephensi* and *Aedes aegypti*

The amount of glucose in the laboratory-reared and field collected aquatic and terrestrial stages of *An. stephensi* and *Ae. aegypti* are shown in Fig. 3. When the glucose content in the laboratory-reared and field collected *An. stephensi* was compared, level of glucose in the field collected mosquitoes was significantly higher than the laboratory-reared mosquitoes (ANOVA, F= 14.24, df= 1, 3, P= 0.03). The level of glucose content in the field collected larvae, pupae, females, and males were 30.49, 93.26, 67.12 and 46.6μg higher than the laboratory-reared mosquitoes respectively (Fig. 3). The glucose content in the laboratory-reared larvae and males of *Ae. aegypti* was 140.94 and 14.43μg lower than the field-collected mosquitoes. However, the glucose content in the field collected pupae and females of *Ae. aegypti* were 9.3 and 110.18μg lower than the conspecific laboratory reared *Ae. aegypti*.

## Discussion

The benefit of the laboratory experiments was to estimate energy reserves to guide the implementation of intervention because it is essential to know how closely the physiology and behavior of laboratory maintained individual represent those from the wild. Much of the information on vector biology and vector control in India comes from the terrestrial land and laboratory experimentation. Therefore, we have recorded the energy reserves of the vectors transmitting malaria and dengue in the desert part of India. The lipid content of the field collected (66.8μg) and laboratory maintained (48.3μg) female *Ae. aegypti* was 196 and 38.2 μg lower in the present study than the value recorded already (29). Wherein, the glycogen (482.87μg) and glucose (182.3μg) content of laboratory maintained female *Ae. aegypti* was 419.07μg and 118.98μg higher in the present case than the amount presented elsewhere (29). The amount of glycogen, glucose, and lipid was measured in female *Ae. aegypti* after 72h of feeding on 10% sucrose solution and recorded the values as 62.4, 42.2 and 95.9μg respectively (19). However, the glycogen (482.87μg) and glucose (182.3μg) content in the laboratory-reared *Ae. aegypti* in the present case was 420.47 and 140μg higher than another value recorded (19) and 47μg lower lipid content was recorded in the present study. The development of insects is heavily regulated by climate and other environmental variables and also vary substantially in response to subtle difference in diet. Variations in energy reserves between the field collected and laboratory-reared mosquitoes may reveal the acquisition of resources and their subsequent assimilation by the larval stages developing in the respective larval habitats under natural conditions, occurred in the present study (30). The variations in the energy contents in the mosquitoes may be due to the habitat and adaptations of the mosquitoes. The energy reserves (glycogen, sugar, and lipid) were compared in four mosquito species in Kolkota, India and recorded higher energy level in females of *Ae. aegypti* than males (30). This observation is similar to the present study except in the case of glucose level in field-collected male. The glycogen level of both the sexes of *Ae. aegypti* (male: 0.221mg, female: 0.223 mg) and lipid level (0.038mg) of male *Ae. aegypti* recorded by (30) were similar to the observation made in the field collected *Ae. aegypti* of the present study.

The lipid, glucose and glycogen in the *An. stephensi* mosquitoes fed were measured with 10% glucose solution and recorded the values in laboratory-reared mosquitoes as 97.3, 101.9 and 82.2μg respectively (31). When the values are compared with the present study, lipid (50.66) and glucose (94.65) content in the present study was 46.64μg and 7.25μg lower than the values recorded by (31). The nutritional reserve of laboratory reserved and field collected male *Anopheles gambiae* and recorded a 5μg high lipid level in the field collected male *An. gambiae* than the laboratory-reared one (32). We have recorded a 2.7μg higher lipid in the laboratory-reared males (34.9 μg) than the field collected males (32.2μg). This may occurred because both the species responded in a similar fashion to the different larval diets (33). The survival and reproductive strategies of the female mosquito differ from the male in many senses, at least one of related to egg production and maturation (34). Dog biscuit is providing carbohydrates and lipid to the larvae and yeast is imparting protein to the larvae. The viability of larva and pupa does not depend upon the carbohydrates, however growth and molting of the larvae is largely dependent upon the protein source (14). The laboratory-reared larvae were provided the food routinely so they can molt from one stage to other. After the introduction of the Indira Gandhi canal in to the desert of Rajasthan, the water retention has been increased in this desert region. This leads to the establishment of mosquito colonization. Mosquitoes get breeding habitat throughout the year because of various water retention. The surrounding of the desert climate provides better habits for mosquito breeding. The field-collected mosquitoes were with more energy may be due to the presence of open water habitats that provide significantly more micro-invertebrate dietary resources for the larvae. Nutrient reserves of adult mosquitoes obtained during the larval stage plays important role in the longevity of mosquitoes. Larvae supplied with the high food amount resulted in adults with higher longevity.

The cases of malaria and dengue are increased continuously in this region after inception of the Indira Gandhi Canal; therefore, physiology of the mosquito should be investigated and compared with insecticide resistance and also with the incrimination of malaria parasite.

## Conclusion

Malaria and dengue in the desert part of India are mainly transmitted by *An. stephensi* and *Ae. aegypti* respectively. Energy content of the mosquitoes was compared between two rearing habitats. The lipid content of laboratory-reared *An. stephensi* and *Ae. aegypti* was found to be lower than their conspecific field-collected specimens. The glycogen content in the laboratory-reared larvae, pupae and female mosquitoes of both the types were higher than that of their conspecific field-collected specimens. The glucose content of the laboratory-reared *An. stephensi* was lower than that of the conspecific field-collected specimens. The higher amount of lipid and lower amount of glycogen in field-collected mosquitoes may be because of the availability of food in the natural habitat and adaptation of mosquitoes. The higher amount of lipid in the field collected mosquitoes suggests these are physiologically better equipped to sustain in the desert ecology.
